# Decline in Vaccination Coverage by Age 24 Months and Vaccination Inequities Among Children Born in 2020 and 2021 — National Immunization Survey-Child, United States, 2021–2023

**DOI:** 10.15585/mmwr.mm7338a3

**Published:** 2024-09-26

**Authors:** Holly A. Hill, David Yankey, Laurie D. Elam-Evans, Yi Mu, Michael Chen, Georgina Peacock, James A. Singleton

**Affiliations:** 1 Immunization Services Division, National Center for Immunization and Respiratory Diseases, CDC.

**Summary**

**What is already known about this topic?**

The Advisory Committee on Immunization Practices currently recommends routine vaccination against 15 potentially serious illnesses for children by age 24 months.

**What is added by this report?**

Estimated coverage with most childhood vaccines was lower among children born during 2020–2021 (during or after the height of the health care disruption from the COVID-19 pandemic) compared with those born during 2018–2019. Disparities by race and ethnicity, health insurance status, poverty status, and urbanicity persist. Coverage also varied widely by jurisdiction, especially for influenza vaccine.

**What are the implications for public health practice?**

Financial barriers, access issues, vaccine hesitancy, and vaccine-related misinformation all need to be overcome to increase coverage, ensure full recovery from the impact of the COVID-19 pandemic, eliminate disparities, and protect all children from vaccine-preventable diseases.

## Abstract

Data from the National Immunization Survey-Child (NIS-Child) were analyzed to estimate coverage with childhood vaccines recommended by the Advisory Committee on Immunization Practices among U.S. children by age 24 months. Coverage with nearly all vaccines was lower among children born in 2020 and 2021 than it was among those born in 2018 and 2019, with declines ranging from 1.3 to 7.8 percentage points. Analyses of NIS-Child data for earlier birth cohorts have not revealed such widespread declines in routine childhood vaccination coverage. Coverage among children born during 2020–2021 varied by race and ethnicity, health insurance status, poverty status, urbanicity, and jurisdiction. Compared with non-Hispanic White children, coverage with four of the 17 vaccine measures was lower among non-Hispanic Black or African American children as well as Hispanic or Latino (Hispanic) and non-Hispanic American Indian or Alaska Native children. Coverage was also generally lower among those covered by Medicaid or other nonprivate insurance, uninsured children, children living below the federal poverty level, and children living in rural areas. Coverage varied widely by jurisdiction, especially coverage with ≥2 doses of influenza vaccine. Children born during 2020–2021 were born during or after the period of major disruption of primary care from the COVID-19 pandemic. Providers should review children’s histories and recommend needed vaccinations during every clinical encounter. Addressing financial barriers, access issues, vaccine hesitancy, and vaccine-related misinformation can also help to increase coverage, reduce disparities, and protect all children from vaccine-preventable diseases. Strategies that have been found effective include implementation of standing orders and reminder and recall systems, strong physician recommendations to vaccinate, and use of immunization information systems to identify areas of lower coverage that could benefit from targeted interventions to increase immunization rates.

## Introduction

The Vaccine National Strategic Plan* has as its vision the elimination of vaccine-preventable diseases from the United States through safe and effective vaccination. The Advisory Committee on Immunization Practices (ACIP) currently recommends routine vaccination against 15 potentially serious illnesses for children by age 24 months (*1*). Since 1994, the National Immunization Survey-Child (NIS-Child) has monitored coverage with ACIP-recommended childhood vaccines.^†^ NIS-Child data are used to calculate annual vaccination coverage estimates at the national and state levels, with additional estimates for some local areas (e.g., cities and counties) and three U.S. territories (Guam, Puerto Rico, and the U.S. Virgin Islands).^§^ This report assesses trends in vaccination coverage by year of birth and disparities in coverage by sociodemographic characteristics. In addition, this report provides a first look at children born in 2021 (during the COVID-19 pandemic) and reaching age 24 months toward or after the end of the COVID-19 public health emergency.

## Methods

### Data Collection

NIS-Child uses random-digit–dialing to identify U.S. households that include a child aged 19–35 months, and interviews are conducted via mobile telephone^¶^ with the parent or guardian (parent) most knowledgeable about the child’s vaccination history. With parental consent, a questionnaire is mailed to each of the child’s vaccine providers to obtain detailed information about vaccines received since birth. Provider-reported data are then synthesized to create a comprehensive vaccination history for each child. For the most recent survey year (children identified in 2023), the household interview response rate** was 27.0%, and adequate provider data^††^ were available for 48.1% of children with completed interviews. Children born during 2020–2021 were identified using data collected during 2021–2023; a total of 28,688 subjects were available for analysis.

### Data Analysis

All coverage estimates in this report are based upon information supplied by vaccination providers. Data were analyzed by birth cohort (year of birth), and for most vaccines, Kaplan-Meier techniques were used to estimate coverage by age 24 months. Exceptions include the birth dose of hepatitis B vaccine (HepB), assessed during the first 3 days of life, and the rotavirus vaccine series, which should be completed by age 8 months. Because of a change in ACIP recommendations in 2020 and a long period of eligibility for catch-up vaccination, coverage with ≥2 doses of hepatitis A vaccine (HepA) was estimated by age 35 months (the maximum age within the scope of NIS-Child data collection) as well as by age 24 months.^§§^ Differences in coverage estimates were evaluated using z-tests at an α-level of 0.05. Nationally and by jurisdiction, estimated coverage among children born in 2020 and 2021 was compared with estimated coverage among children born in 2018 and 2019. For data stratified by sociodemographic characteristics, subgroup estimates were compared with those for a designated referent group. Analyses used weighted data and were performed using SAS software (version 9.4; SAS Institute) and SUDAAN software (version 11; RTI International). This activity was reviewed by CDC, deemed not research, and was conducted consistent with applicable federal law and CDC policy.^¶¶^

## Results

### Recent Trends in National Vaccination Coverage by Birth Year

Estimated coverage with all recommended childhood vaccines was lower among children born in 2020 and 2021 than among those born in 2018 and 2019, except for the HepB birth dose and ≥2 doses of HepA (Table 1). Most decreases ranged from 1.3 percentage points for ≥1 dose of varicella vaccine to 3.2 percentage points for the full series of *Haemophilus influenzae* type b conjugate vaccine (Hib) and the combined seven-vaccine series,*** with a larger (7.8 percentage point) drop in coverage with ≥2 doses of influenza vaccine. Longer term trends by single-year birth cohort reveal decreases in coverage with ≥2 doses of influenza vaccine from the 2019 to 2020 birth cohort and from the 2020 to 2021 birth cohort (Supplementary Figure, https://stacks.cdc.gov/view/cdc/162212). Aside from declines in influenza vaccine coverage, the largest observed declines in coverage were for those vaccines and vaccine doses with recommended series completion during the second year of life (i.e., the fourth dose of diphtheria and tetanus toxoids, and acellular pertussis vaccine [DTaP], the final dose in the full Hib series, and the fourth dose of pneumococcal conjugate vaccine [PCV]). Despite the decreases, coverage with several vaccines remained above 90%, including poliovirus vaccine (91.9%); ≥1 dose of measles, mumps, and rubella vaccine (MMR) (90.3%); and ≥3 doses of HepB (91.1%) (Table 1). The lowest estimated coverage was with ≥2 doses of HepA by 24 months (46.0%), and ≥2 doses of influenza vaccine (55.6%). The percentage of children who received no vaccinations by age 24 months remained low (1.2%).

**TABLE 1:** Estimated vaccination coverage, by age 24 months* among children born during 2018–2019 and 2020–2021 for selected vaccines and doses — National Immunization Survey-Child, United States, 2019–2023

Vaccine/Dose	% (95% CI)		
	Birth year^†^		Difference (2018–2019 to 2020–2021)
	2018–2019	2020–2021	
**DTaP^§^**			
≥3 doses	94.3 (93.8 to 94.7)	92.5 (91.8 to 93.2)	−1.8 (−2.6 to −1.0)^¶^
≥4 doses	81.8 (81.0 to 82.6)	79.3 (78.2 to 80.4)	−2.5 (−3.8 to −1.1)^¶^
**Poliovirus (≥3 doses)**	93.4 (92.9 to 93.9)	91.9 (91.2 to 92.6)	−1.5 (−2.4 to −0.6)^¶^
MMR (≥1 dose)**	92.0 (91.4 to 92.6)	90.3 (89.6 to 91.0)	−1.7 (−2.6 to −0.7)^¶^
**Hib^††^**			
Primary series	93.8 (93.3 to 94.3)	91.6 (90.8 to 92.3)	−2.2 (−3.1 to −1.3)^¶^
Full series	80.0 (79.2 to 80.9)	76.8 (75.7 to 77.9)	−3.2 (−4.6 to −1.8)^¶^
**HepB**			
Birth dose^§§^	80.3 (79.4 to 81.1)	79.5 (78.5 to 80.5)	−0.8 (−2.1 to 0.6)
≥3 doses	92.6 (92.0 to 93.1)	91.1 (90.3 to 91.8)	−1.5 (−2.4 to −0.6)^¶^
VAR (≥1 dose)**	91.2 (90.5 to 91.8)	89.9 (89.1 to 90.6)	−1.3 (−2.2 to −0.3)^¶^
**PCV**			
≥3 doses	93.4 (92.9 to 93.9)	91.6 (90.9 to 92.3)	−1.8 (−2.6 to −0.9)^¶^
≥4 doses	83.4 (82.7 to 84.2)	80.7 (79.6 to 81.8)	−2.7 (−4.1 to −1.4)^¶^
**HepA** ^¶¶^			
≥1 dose	88.1 (87.4 to 88.8)	86.5 (85.6 to 87.4)	−1.6 (−2.7 to −0.4)^¶^
≥2 doses (by age 24 mos)	47.6 (46.5 to 48.6)	46.0 (44.8 to 47.2)	−1.6 (−3.2 to 0)
≥2 doses (by age 35 mos)	79.8 (78.6 to 81.0)	77.7 (76.1 to 79.2)	−2.1 (−4.1 to −0.1)^¶^
**Rotavirus** (by age 8 mos)***	77.1 (76.2 to 78.0)	75.1 (74.0 to 76.2)	−2.0 (−3.4 to −0.5)^¶^
**Influenza (≥2 doses)^†††^**	63.4 (62.4 to 64.4)	55.6 (54.4 to 56.8)	−7.8 (−9.4 to −6.2)^¶^
**Combined seven-vaccine series^§§§^**	70.1 (69.2 to 71.1)	66.9 (65.7 to 68.2)	−3.2 (−4.8 to −1.6)^¶^
**No vaccinations^¶¶¶^**	0.9 (0.8 to 1.1)	1.2 (1.0 to 1.4)	0.2 (0 to 0.5)

**Abbreviations:** DTaP = diphtheria, tetanus toxoids, and acellular pertussis vaccine; HepA = hepatitis A vaccine; HepB = hepatitis B vaccine; Hib = *Haemophilus influenzae* type b conjugate vaccine; MMR = measles, mumps, and rubella vaccine; PCV = pneumococcal conjugate vaccine; VAR = varicella vaccine.

* Includes vaccinations received by age 24 months (before the day the child turns age 24 months), except for the HepB birth dose (at birth through age 3 days), rotavirus vaccination (by age 8 months), and ≥2 HepA doses (by age 35 months). For all vaccines except the HepB birth dose and rotavirus vaccination, the Kaplan-Meier method was used to estimate vaccination coverage to account for children whose vaccination history was ascertained before age 24 months (also at age 35 months for ≥2 HepA doses).

^†^ Data for the 2018 birth year are from survey years 2019, 2020, and 2021; data for the 2019 birth year are from survey years 2020, 2021, and 2022; data for the 2020 birth year are from survey years 2021, 2022, and 2023; data for the 2021 birth year are considered preliminary and come from survey years 2022 and 2023 (data from survey year 2024 are not yet available).

^§^ Includes children who might have been vaccinated with diphtheria and tetanus toxoids vaccine or diphtheria, tetanus toxoids, and pertussis vaccine. Healthy People 2030 target for ≥4 doses of DTaP by age 2 years is 90%. https://health.gov/healthypeople/objectives-and-data/browse-objectives/vaccination/increase-coverage-level-4-doses-dtap-vaccine-children-age-2-years-iid-06

^¶^ Statistically significantly different (p<0.05) from zero.

** Includes children who might have been vaccinated with measles, mumps, rubella, and varicella combination vaccine. Healthy People 2030 target for ≥1 dose of MMR by age 2 years is 90.8%. https://health.gov/healthypeople/objectives-and-data/browse-objectives/vaccination/maintain-vaccination-coverage-level-1-dose-mmr-vaccine-children-age-2-years-iid-03

^††^ Hib primary series: receipt of ≥2 or ≥3 doses, depending on product type received; full series: primary series and booster dose, which includes receipt of ≥3 or ≥4 doses, depending on product type received.

^§§^ One dose of HepB administered from birth through age 3 days.

^¶¶^ Before 2020, the first dose of HepA was recommended at age 12–23 months, with the second dose given 6–18 months after the first, depending upon the product type received. In 2020, the recommendation was revised to 2 doses between ages 12 and 23 months, ≥6 months apart. Because children in this analysis were vaccinated under both recommendations, coverage estimates for both age <24 months and age <35 months are provided.

*** Includes ≥2 doses of Rotarix (GSK) monovalent rotavirus vaccine, or ≥3 doses of RotaTeq (Merck & Co., Inc.) pentavalent rotavirus vaccine. (If any dose in the series is either RotaTeq or unknown, defaults to the 3-dose series.) The maximum age for the final rotavirus dose is 8 months, 0 days.

^†††^ Doses must be ≥24 days apart (4 weeks with a 4-day grace period); doses could have been received during two influenza seasons.

^§§§^ The combined seven-vaccine series (4:3:1:3*:3:1:4) includes ≥4 doses of DTaP, ≥3 doses of poliovirus vaccine, ≥1 dose of measles-containing vaccine, the full series of Hib (≥3 or ≥4 doses, depending on product type), ≥3 doses of HepB, ≥1 dose of VAR, and ≥4 doses of PCV.

^¶¶¶^ Healthy People 2030 target for children who receive no recommended vaccines by age 2 years is ≤1.3%. https://health.gov/healthypeople/objectives-and-data/browse-objectives/vaccination/reduce-proportion-children-who-get-no-recommended-vaccines-age-2-years-iid-02

### Vaccination Coverage by Selected Sociodemographic Characteristics and Jurisdictions

Disparities in coverage by race and ethnicity were observed among children born during 2020–2021 (Table 2). Coverage with ≥4 doses of DTaP, ≥4 doses of PCV, rotavirus vaccine, and the combined seven-vaccine series was lower among non-Hispanic Black or African American (Black) children, Hispanic or Latino (Hispanic) children, and non-Hispanic American Indian or Alaska Native (AI/AN) children than among non-Hispanic White (White) children. Compared with coverage among White children, coverage with ≥2 doses of influenza vaccine was lower among Black and Hispanic children but higher among non-Hispanic Asian children.

**TABLE 2:** Estimated vaccination coverage, by age 24 months* among children born during 2020–2021,^†^ by selected vaccines and doses and race and ethnicity^§^ — National Immunization Survey–Child, United States, 2021–2023

Vaccine/Dose	Race and ethnicity,^§^ % (95% CI)						
	White (referent) n = 16,656	Black or African American n = 2,324	Hispanic or Latino n = 5,331	AI/AN n = 316	Asian n = 1,460	NH/PI n = 89	Multiple races n = 2,492
**DTaP^¶^**							
≥3 doses	92.8 (91.8–93.6)	92.1 (90.0–94.0)	92.9 (91.5–94.1)	87.9 (82.0–92.6)	92.8 (89.2–95.6)	88.0 (76.6–95.5)	90.8 (88.1–93.2)
≥4 doses	81.4 (80.1–82.6)	76.1 (72.3–79.6)**	77.5 (74.8–80.0)**	72.4 (64.2–80.1)**	83.1 (78.8–86.9)	—^††^	78.5 (75.1–81.8)
**Poliovirus (≥3 doses)**	92.2 (91.2–93.1)	91.6 (89.3–93.5)	92.1 (90.5–93.5)	88.9 (83.2–93.3)	92.2 (88.6–95.1)	88.0 (76.6–95.5)	90.3 (87.6–92.6)
**MMR (≥1 dose)^§§^**	90.2 (89.2–91.1)	89.2 (87.1–91.2)	91.3 (89.7–92.7)	87.7 (81.5–92.7)	91.8 (88.4–94.6)	—^††^	88.8 (85.8–91.4)
**Hib^¶¶^**							
Primary series	91.8 (90.7–92.8)	91.0 (88.8–93.0)	92.2 (90.8–93.4)	86.1 (79.2–91.6)	91.5 (87.8–94.4)	87.0 (75.9–94.6)	89.9 (87.0–92.5)
Full series	77.9 (76.5–79.3)	73.7 (70.1–77.3)**	76.1 (73.6–78.5)	72.2 (64.0–79.9)	80.6 (76.5–84.3)	—^††^	76.4 (72.9–79.8)
**HepB**							
Birth dose***	79.3 (78.0–80.6)	77.6 (74.3–80.6)	79.7 (77.3–81.9)	78.6 (69.4–85.6)	81.7 (77.6–85.2)	—^††^	82.0 (78.6–85.0)
≥3 doses	91.0 (90.0–91.9)	91.2 (89.0–93.2)	91.3 (89.5–92.9)	90.6 (85.4–94.4)	91.6 (88.7–94.1)	—^††^	90.0 (87.4–92.4)
**VAR (≥1 dose)^§§^**	89.8 (88.7–90.7)	90.0 (88.0–91.8)	91.0 (89.3–92.4)	83.0 (74.6–89.9)	88.6 (84.8–91.9)	89.1 (77.9–96.1)	88.1 (85.0–90.8)
**PCV**							
≥3 doses	91.7 (90.7–92.7)	91.3 (89.0–93.3)	92.5 (91.2–93.7)	85.1 (77.2–91.4)	90.8 (87.1–93.8)	87.4 (75.9–95.1)	90.0 (87.1–92.5)
≥4 doses	83.1 (81.8–84.3)	77.9 (74.2–81.4)**	78.4 (76.0–80.8)**	74.2 (65.6–82.1)**	83.0 (79.0–86.7)	—^††^	80.2 (76.9–83.3)
**HepA^†††^**							
≥1 dose	86.4 (85.2–87.5)	83.9 (80.4–87.1)	87.5 (85.4–89.4)	81.9 (74.7–88.1)	88.6 (85.1–91.6)	—^††^	87.2 (84.2–89.9)
≥2 doses (by age 24 mos)	47.3 (45.8–48.8)	40.5 (37.1–44.2)**	45.8 (43.0–48.7)	40.9 (32.2–51.0)	49.5 (44.9–54.3)	—^††^	47.6 (43.7–51.7)
≥2 doses (by age 35 mos)	79.8 (78.0–81.6)	72.7 (67.4–77.8)**	76.1 (72.6–79.4)	—^††^	81.2 (74.5–87.0)	—^††^	79.1 (74.0–83.9)
**Rotavirus (by age 8 mos)^§§§^**	77.3 (76.0–78.6)	71.4 (67.7–74.7)**	72.9 (70.3–75.4)**	67.2 (57.6–75.5)**	79.7 (75.6–83.3)	—^††^	75.5 (71.6–79.0)
**Influenza ≥2 doses^¶¶¶^**	59.6 (58.0–61.1)	42.6 (39.0–46.3)**	52.8 (49.9–55.7)**	51.5 (42.7–61.1)	71.4 (66.9–75.8)**	—^††^	55.6 (51.8–59.5)
Combined seven-vaccine series****	68.8 (67.3–70.2)	64.0 (60.2–67.8)**	65.0 (62.1–67.8)**	58.7 (49.7–67.9)**	70.0 (65.4–74.5)	—^††^	67.9 (64.2–71.6)
**No vaccinations**	1.4 (1.2–1.7)	0.9 (0.6–1.3)**	1.0 (0.7–1.4)	—^††^	—^††^	—^††^	—^††^

**Abbreviations:** AI/AN = American Indian or Alaska Native; DTaP = diphtheria, tetanus toxoids, and acellular pertussis vaccine; HepA = hepatitis A vaccine; HepB = hepatitis B vaccine; Hib = *Haemophilus influenzae* type b conjugate vaccine; MMR = measles, mumps, and rubella vaccine; NH/PI = Native Hawaiian or Pacific Islander; PCV = pneumococcal conjugate vaccine; VAR = varicella vaccine.

* Includes vaccinations received by age 24 months (before the day the child turns age 24 months), except for the HepB birth dose (at birth through age 3 days), rotavirus vaccination (by age 8 months), and ≥2 HepA doses (by age 35 months). For all vaccines except the HepB birth dose and rotavirus vaccination, the Kaplan-Meier method was used to estimate vaccination coverage to account for children whose vaccination history was ascertained before age 24 months (also at age 35 months for ≥2 HepA doses).

^†^ Data for the 2020 birth year are from survey years 2021, 2022, and 2023; data for the 2021 birth year are considered preliminary and come from survey years 2022 and 2023 (data from survey year 2024 are not yet available).

^§^ Children’s race and ethnicity was reported by the parent or guardian. Children identified in this report as AI/AN, Asian, Black or African American, NH/PI, White, or multiple races were reported by the parent or guardian as non-Hispanic. Children identified as being of multiple races had more than one race category selected. Children identified as Hispanic or Latino (Hispanic) might be of any race.

^¶^ Includes children who might have been vaccinated with diphtheria and tetanus toxoids vaccine or diphtheria, tetanus toxoids, and pertussis vaccine.

** Statistically significant (p<0.05) difference compared with the referent group.

^††^ Estimate not available because the unweighted sample size for the denominator was <30, or 95% CI half width / estimate >0.588, or 95% CI half-width was ≥10.

^§§^ Includes children who might have been vaccinated with measles, mumps, rubella, and varicella combination vaccine.

^¶¶^ Hib primary series: receipt of ≥2 or ≥3 doses, depending on product type received; full series: primary series and booster dose, which includes receipt of ≥3 or ≥4 doses, depending on product type received.

*** One dose of HepB administered from birth through age 3 days.

^†††^ Before 2020, the first dose of HepA was recommended at age 12–23 months, with the second dose given 6–18 months after the first, depending upon the product type received. In 2020, the recommendation was revised to 2 doses between ages 12 and 23 months, ≥6 months apart. Because children in this analysis were vaccinated under both recommendations, coverage estimates for both age <24 months and age <35 months are provided.

^§§§^ Includes ≥2 doses of Rotarix (GSK) monovalent rotavirus vaccine, or ≥3 doses of RotaTeq (Merck & Co., Inc.) pentavalent rotavirus vaccine. (If any dose in the series is either RotaTeq or unknown, defaults to the 3-dose series.) The maximum age for the final rotavirus dose is 8 months, 0 days.

^¶¶¶^ Doses must be ≥24 days apart (4 weeks with a 4-day grace period); doses could have been received during two influenza seasons.

**** The combined seven-vaccine series (4:3:1:3*:3:1:4) includes ≥4 doses of DTaP, ≥3 doses of poliovirus vaccine, ≥1 dose of measles-containing vaccine, the full series of Hib (≥3 or ≥4 doses, depending on product type), ≥3 doses of HepB, ≥1 dose of VAR, and ≥4 doses of PCV.

By health insurance status, coverage with all vaccines was lower among children who were covered by Medicaid or other nonprivate insurance, and those who were uninsured, than among those covered solely by private insurance (Supplementary Table 1, https://stacks.cdc.gov/view/cdc/162213). Similarly, coverage with all vaccines was lower among children living below the federal poverty level than among those living at or above the poverty level, with percentage point differences ranging from 2.7 (≥1 dose of MMR) to 19.9 (≥2 doses of influenza vaccine) (Supplementary Table 2, https://stacks.cdc.gov/view/cdc/162214). No differences in coverage were observed between children living in a metropolitan statistical area (MSA)^†††^ principal city and those living in an MSA nonprincipal city. However, coverage was lower among children living in a non-MSA compared with an MSA principal city for all vaccines except the full Hib series, the HepB birth dose, and ≥2 doses of HepA. Substantial jurisdictional variation in coverage estimates was also observed with selected vaccines (Table 3), especially ≥2-dose influenza vaccination coverage, which ranged from 25.6% in Mississippi to 80.3% in Rhode Island. Comparing the 2018–2019 and 2020–2021 birth cohorts by jurisdiction for each of eight vaccine measures identified 69 statistically significant differences in coverage estimates, 64 (92.8%) of which reflected lower vaccination coverage among the more recent (2020–2021) birth cohorts.

**TABLE 3:** Estimated vaccination coverage with selected individual vaccines and a combined vaccine series,* by age 24 months^†^ among children born during 2020–2021,^§^ by U.S. Department of Health and Human Services region, state, selected local area, and territory — National Immunization Survey-Child, United States, 2021–2023

U.S. national, HHS region, state or local area, or territory	No.	Vaccine/Vaccine series, % (95% CI)							
		MMR^¶^ (≥1 dose)	Poliovirus	DTaP** (≥4 doses)	HepB^††^ (birth dose)	HepA (≥2 doses by age 35 mos)	Rotavirus^§§^	Influenza^¶¶^ (≥2 doses)	Combined seven-vaccine series*
**U.S. national**	28,668	90.3 (89.6–91.0)***	91.9 (91.2–92.6)***	79.3 (78.2–80.4)***	79.5 (78.5–80.5)	77.7 (76.1–79.2)***	75.1 (74.0–76.2)***	55.6 (54.4–56.8)***	66.9 (65.7–68.2)***
**HHS Region 1**	2,578	94.9 (92.9–96.5)	96.4 (95.1–97.5)	87.8 (85.1–90.2)	83.8 (80.7–86.6)	90.0 (85.3–93.6)	85.8 (83.4–88.0)	75.0 (71.3–78.5)***	79.6 (76.3–82.7)
Connecticut	412	94.4 (90.4–97.2)	95.4 (91.4–97.9)	86.7 (81.2–91.3)	83.9 (78.1–88.3)	91.2 (83.6–96.2)	84.5 (78.9–88.9)	79.8 (73.7–85.3)	76.7 (70.5–82.5)
Maine	366	94.2 (90.7–96.7)	95.5 (92.5–97.5)	86.1 (81.0–90.5)	82.8 (77.3–87.2)	80.2 (72.6–86.8)	75.8 (69.6–81.0)	73.0 (66.8–78.8)	74.4 (68.4–80.0)
Massachusetts	443	94.9 (91.0–97.5)	97.1 (94.8–98.6)	88.5 (83.6–92.4)	85.2 (78.9–89.8)	93.8 (83.7–98.6)	89.4 (85.1–92.6)^†††^	72.4 (65.7–78.8)***	83.1 (77.0–88.4)
New Hampshire	315	95.5 (92.6–97.6)	96.5 (93.6–98.3)	89.4 (84.4–93.4)	81.4 (75.3–86.2)	78.9 (70.4–86.4)	80.8 (74.6–85.7)	72.5 (66.1–78.5)	77.2 (70.9–82.9)
Rhode Island	542	95.8 (93.1–97.7)	96.3 (93.4–98.2)	88.3 (84.1–91.7)	81.6 (76.6–85.8)	90.0 (82.4–95.2)	86.2 (81.4–89.9)	80.3 (75.4–84.8)	77.8 (72.8–82.3)
Vermont	500	95.2 (91.7–97.6)	95.6 (92.0–97.8)	86.4 (81.6–90.5)	78.6 (73.1–83.2)	84.9 (76.6–91.5)	82.0 (76.9–86.2)	75.7 (70.0–81.0)	75.6 (69.9–81.0)
**HHS Region 2**	1,462	89.6 (86.8–92.0)	92.8 (90.5–94.7)	82.1 (78.8–85.1)	78.0 (74.8–80.9)	73.7 (68.5–78.7)	74.8 (71.4–77.9)	62.1 (58.4–65.7)***	67.5 (64.0–71.1)
New Jersey	436	89.3 (83.6–93.7)	93.4 (89.2–96.3)	83.0 (76.8–88.4)	81.5 (76.0–86.0)	76.9 (67.0–85.5)	75.0 (68.6–80.4)	64.5 (58.0–70.9)***	63.7 (57.0–70.3)
New York	1,026	89.7 (86.6–92.3)***	92.5 (89.6–94.8)	81.6 (77.7–85.2)	76.3 (72.3–79.8)	72.2 (66.1–78.0)	74.7 (70.6–78.4)	60.8 (56.4–65.2)***	69.5 (65.3–73.6)
New York City	560	86.7 (82.1–90.6)***	92.0 (87.8–95.1)	80.7 (75.3–85.6)	72.8 (67.1–77.8)	68.7 (59.9–77.1)	73.3 (67.5–78.4)	62.2 (56.2–68.2)	68.0 (62.1–73.8)
New York, excluding New York City	466	92.1 (87.7–95.4)	92.9 (88.6–96.0)	82.3 (76.7–87.3)	79.2 (73.4–84.0)	75.0 (66.6–82.7)	75.9 (69.9–81.0)	59.6 (53.3–66.0)***	70.8 (64.8–76.5)
**HHS Region 3**	3,970	89.9 (88.0–91.6)***	92.1 (90.5–93.6)	80.5 (78.1–82.7)	80.0 (77.7–82.1)***	79.6 (76.2–82.8)	77.7 (75.3–79.9)	62.5 (59.8–65.2)***	69.0 (66.5–71.6)***
Delaware	293	91.0 (86.3–94.6)	90.5 (85.6–94.3)	81.1 (74.8–86.6)	80.7 (74.2–85.9)	84.9 (75.2–92.3)	71.1 (63.7–77.5)	58.7 (51.3–66.2)***	68.1 (60.9–75.1)
District of Columbia	463	88.4 (83.0–92.7)	90.1 (85.2–94.0)	80.3 (73.7–86.1)	78.2 (71.6–83.6)	74.5 (64.8–83.3)	75.9 (69.0–81.6)	67.9 (60.5–75.2)	71.2 (64.3–77.9)
Maryland	861	95.4 (93.0–97.1)	96.0 (93.7–97.7)	87.3 (83.7–90.5)	80.0 (75.6–83.8)	86.5 (80.9–91.1)	82.0 (77.9–85.5)	66.5 (61.9–71.1)	75.7 (71.4–79.9)
Pennsylvania	1,121	88.6 (85.0–91.6)***	91.2 (88.2–93.7)	77.9 (73.5–82.1)	83.5 (79.6–86.8)	78.8 (72.4–84.6)	79.0 (74.8–82.6)	61.7 (56.6–66.8)***	67.1 (62.4–71.9)***
Philadelphia	546	89.7 (85.2–93.3)	88.8 (84.2–92.6)	76.3 (70.4–81.9)	82.2 (76.8–86.5)	79.7 (71.1–87.1)	75.0 (69.0–80.1)	62.9 (56.7–69.0)	65.5 (59.2–71.7)
Pennsylvania, excluding Philadelphia	575	88.4 (84.3–91.9)***	91.6 (88.1–94.4)	78.2 (73.1–83.0)	83.8 (79.2–87.5)	78.7 (71.3–85.3)	79.7 (74.8–83.8)	61.5 (55.7–67.3)***	67.4 (61.9–72.9)
Virginia	804	88.0 (84.1–91.4)	91.6 (88.0–94.5)	79.8 (75.1–84.2)	75.2 (70.3–79.6)	75.3 (68.8–81.4)	74.7 (69.3–79.4)	63.7 (58.5–68.9)***	67.8 (62.8–72.7)***
West Virginia	428	87.6 (82.4–91.9)	88.3 (83.4–92.3)***	74.2 (68.2–79.9)	80.0 (74.2–84.7)	76.6 (69.7–83.0)	72.3 (65.8–78.0)	46.5 (40.6–52.9)	63.2 (57.0–69.4)
**HHS Region 4**	4,478	89.7 (88.2–91.2)***	92.2 (90.8–93.5)	79.6 (77.5–81.6)	78.5 (76.5–80.5)	76.3 (73.0–79.5)	72.7 (70.4–74.8)	43.0 (40.6–45.4)***	66.0 (63.6–68.3)***
Alabama	400	89.8 (84.6–93.8)	89.2 (83.8–93.4)	79.7 (73.6–85.2)	76.7 (70.3–82.1)	75.3 (66.9–82.9)	69.8 (62.6–76.1)	33.4 (27.2–40.5)	62.6 (55.7–69.5)
Florida	666	90.8 (87.2–93.8)	91.6 (87.9–94.5)	80.1 (75.0–84.8)	76.3 (71.1–80.8)	76.1 (67.0–84.2)	70.9 (65.3–75.9)	42.1 (36.5–48.1)	67.3 (61.5–72.9)
Georgia	593	84.3 (79.2–88.7)***	91.5 (87.8–94.5)	76.4 (70.8–81.5)	79.4 (74.1–83.8)	76.0 (67.7–83.5)	70.9 (64.8–76.3)	40.4 (34.6–46.7)	64.3 (58.3–70.3)
Kentucky	540	88.5 (84.0–92.1)	91.9 (87.9–94.9)	79.3 (73.9–84.2)	79.8 (74.8–84.1)	77.6 (70.8–83.7)	69.2 (63.6–74.4)***	45.9 (40.3–51.9)***	64.1 (58.3–69.9)***
Mississippi	505	87.9 (82.6–92.2)	88.0 (82.4–92.4)	74.3 (67.7–80.5)	79.0 (73.1–83.9)	—^§§§^	62.2 (55.3–68.6)	25.6 (20.7–31.3)***	64.8 (58.0–71.6)
North Carolina	764	94.1 (91.6–96.1)	96.6 (94.9–97.9)	81.4 (77.1–85.4)	84.9 (80.9–88.2)	76.5 (70.5–82.0)	80.0 (75.4–83.9)	49.5 (44.3–54.9)***	69.7 (64.7–74.5)
South Carolina	431	88.7 (83.8–92.7)	90.2 (85.5–93.9)	77.7 (71.8–83.1)	80.2 (74.7–84.8)	—^§§§^	78.1 (71.9–83.3)	37.8 (32.2–43.9)	64.5 (58.2–70.6)
Tennessee	579	91.1 (87.0–94.3)	93.9 (90.5–96.5)	83.3 (78.2–87.7)	73.0 (67.1–78.2)	83.9 (74.6–91.2)	74.2 (68.5–79.2)	55.7 (49.7–61.9)***	64.4 (58.4–70.3)
**HHS Region 5**	3,717	91.8 (90.4–93.1)	92.5 (91.1–93.7)	80.7 (78.7–82.7)	81.4 (79.4–83.1)	80.1 (77.1–82.9)	75.8 (73.7–77.8)	60.2 (57.9–62.5)***	68.7 (66.5–70.9)
Illinois	918	92.7 (89.6–95.1)	93.2 (90.4–95.3)	80.0 (75.9–83.8)	79.2 (75.2–82.8)	76.3 (70.1–82.0)	74.9 (70.6–78.8)	58.7 (54.2–63.2)	66.4 (62.0–70.7)
Chicago	280	91.6 (86.9–95.1)	91.9 (86.9–95.5)	81.8 (75.3–87.5)	81.6 (74.6–86.9)	74.2 (64.0–83.4)	75.9 (68.2–82.2)	64.4 (56.7–72.2)	63.9 (56.3–71.5)
Illinois, excluding Chicago	638	93.0 (89.1–95.9)	93.5 (90.2–96.0)	79.5 (74.5–84.1)	78.6 (73.7–82.8)	76.6 (69.3–83.3)	74.6 (69.4–79.2)	57.0 (51.7–62.4)	67.2 (61.9–72.4)
Indiana	369	91.9 (87.7–95.1)	93.5 (89.5–96.4)	81.7 (75.8–86.9)	81.4 (75.2–86.3)	80.1 (70.1–88.4)	72.7 (65.9–78.5)	50.5 (44.0–57.4)***	71.7 (65.3–77.8)
Michigan	771	93.4 (89.9–95.9)	92.4 (89.1–95.1)***	78.6 (73.3–83.5)	78.3 (73.3–82.6)	80.8 (74.4–86.5)***	78.7 (73.8–82.9)	64.4 (58.8–69.9)	67.7 (62.3–73.0)
Minnesota	415	92.0 (87.6–95.3)	92.8 (88.6–95.9)	82.0 (75.9–87.4)	81.3 (75.0–86.4)	—^§§§^	77.2 (70.7–82.6)	68.0 (61.5–74.3)	68.9 (62.4–75.3)
Ohio	759	90.2 (86.9–93.0)	91.5 (88.4–94.1)	82.0 (77.6–86.0)	84.8 (80.9–88.1)	80.3 (74.2–85.7)	74.9 (70.1–79.3)	56.4 (51.3–61.6)***	69.9 (65.1–74.5)
Wisconsin	485	90.9 (86.9–94.1)	91.1 (87.1–94.3)	80.0 (74.7–84.8)	83.8 (78.4–88.0)	85.6 (78.9–91.0)	77.6 (72.0–82.3)	69.1 (63.2–74.8)	68.4 (62.7–74.0)
**HHS Region 6**	3,492	91.7 (89.7–93.4)	91.8 (89.6–93.7)	79.5 (76.1–82.7)	81.6 (78.4–84.4)	78.3 (73.5–82.7)	75.7 (72.0–79.1)	48.7 (44.6–52.9)***	66.6 (62.7–70.5)
Arkansas	513	86.6 (80.9–91.2)	86.5 (81.0–91.1)	73.4 (67.0–79.5)	82.9 (77.5–87.3)	79.4 (71.2–86.6)	73.4 (67.1–78.9)	40.4 (34.0–47.5)	62.0 (55.4–68.6)
Louisiana	697	90.7 (87.1–93.7)	93.0 (89.6–95.6)	81.3 (76.4–85.8)^†††^	76.8 (71.8–81.2)	72.6 (65.0–79.7)	74.3 (68.9–79.1)^†††^	40.8 (35.5–46.6)	68.5 (62.7–74.0)
New Mexico	595	89.6 (85.2–93.2)***	90.1 (85.9–93.5)***	76.7 (71.1–81.8)	74.4 (69.1–79.1)	77.4 (70.7–83.5)	75.1 (69.6–79.9)***	55.4 (49.7–61.3)***	68.2 (62.5–73.7)
Oklahoma	446	88.0 (83.2–92.0)	92.6 (89.0–95.4)	78.2 (72.5–83.4)	77.2 (71.5–82.1)	78.5 (71.3–85.0)	73.9 (68.2–78.9)	44.0 (38.0–50.5)	64.9 (58.7–71.1)
Texas	1,241	92.8 (90.2–95.0)	92.1 (88.9–94.6)	80.0 (75.2–84.3)	83.1 (78.5–86.9)	79.3 (72.2–85.5)	76.4 (71.1–80.9)	50.8 (45.1–56.7)	66.8 (61.4–72.2)
Bexar County	313	86.4 (81.1–90.8)	88.2 (82.8–92.6)	76.9 (70.1–83.0)	73.4 (65.7–80.0)	73.5 (63.7–82.4)	73.1 (65.4–79.6)	47.7 (40.0–56.2)***	62.3 (54.5–70.1)
Houston	339	87.8 (82.1–92.3)	90.8 (85.4–94.9)	73.9 (66.2–81.0)	79.7 (72.9–85.1)	—^§§§^	75.4 (67.7–81.8)	56.8 (49.1–64.8)	65.7 (57.9–73.4)
Texas, excluding Bexar County and Houston	589	94.1 (90.9–96.5)	92.6 (88.7–95.5)	81.2 (75.4–86.3)	84.4 (78.8–88.8)	79.3 (70.8–86.7)	76.8 (70.4–82.2)	50.2 (43.4–57.4)	67.4 (60.8–73.8)
**HHS Region 7**	2,168	91.3 (89.4–92.9)	92.5 (90.8–94.1)	80.6 (77.9–83.2)	82.4 (79.9–84.6)	80.8 (76.8–84.5)	78.7 (76.0–81.2)	54.8 (51.7–57.9)***	67.5 (64.5–70.6)
Iowa	455	90.2 (85.2–94.0)***	92.7 (88.4–95.8)	80.4 (74.1–85.9)***	82.8 (77.1–87.4)	81.3 (71.4–89.3)	79.4 (73.0–84.6)	45.8 (39.4–52.8)	70.9 (64.2–77.3)***
Kansas	634	90.1 (86.2–93.2)	90.4 (86.7–93.4)	77.6 (72.3–82.4)	77.5 (72.1–82.1)	81.7 (73.7–88.4)	75.6 (70.1–80.4)	55.0 (49.5–60.7)	66.4 (60.9–71.9)
Missouri	657	91.7 (88.6–94.3)	92.9 (89.9–95.3)	79.7 (74.9–84.1)	85.0 (81.1–88.2)	80.1 (73.2–86.2)	79.7 (75.2–83.6)^†††^	54.0 (48.7–59.5)	68.6 (63.3–73.8)
Nebraska	422	93.6 (89.5–96.5)	94.7 (91.5–96.9)	88.2 (83.4–92.2)	81.3 (75.0–86.3)	81.9 (74.5–88.1)	79.4 (73.7–84.2)	70.5 (63.9–76.9)	61.2 (54.5–68.0)***
**HHS Region 8**	2,759	88.4 (85.9–90.6)***	90.7 (88.5–92.6)***	78.5 (75.6–81.2)***	81.1 (78.4–83.5)	79.0 (75.0–82.7)	73.7 (70.6–76.6)***	64.0 (60.8–67.2)***	68.0 (64.9–71.1)***
Colorado	482	86.0 (80.8–90.3)***	88.9 (84.1–92.7)***	77.0 (71.4–82.2)	80.5 (75.0–85.0)	78.8 (71.1–85.6)	71.9 (65.8–77.3)***	67.8 (61.9–73.6)	66.1 (60.2–71.9)
Montana	347	86.3 (81.6–90.4)	89.7 (85.4–93.2)	69.6 (63.2–75.7)***	71.7 (65.2–77.3)	62.7 (53.8–71.5)	65.5 (58.8–71.6)***	54.8 (48.3–61.6)***	57.8 (51.2–64.5)***
North Dakota	391	87.0 (81.6–91.5)***	90.3 (86.0–93.7)***	77.3 (70.9–83.1)***	86.9 (81.6–90.9)	79.7 (71.2–87.1)	77.6 (71.0–83.0)	67.3 (60.5–74.0)	71.9 (65.1–78.3)***
South Dakota	483	89.9 (84.9–93.8)	87.8 (82.0–92.4)***	71.9 (64.8–78.6)	81.8 (75.2–86.8)	82.6 (72.3–90.8)	76.6 (69.5–82.4)	62.9 (55.6–70.2)***	63.9 (56.6–71.1)
Utah	559	91.8 (88.2–94.7)	94.1 (91.7–96.0)	84.7 (80.4–88.5)	83.8 (79.3–87.5)	82.8 (77.2–87.7)	76.4 (71.1–81.0)***	62.1 (56.6–67.7)***	74.0 (68.7–79.0)
Wyoming	497	89.6 (84.8–93.4)	90.4 (86.1–93.8)	76.1 (70.0–81.7)	74.5 (68.2–79.8)	69.0 (61.3–76.3)	77.0 (71.1–82.0)	54.3 (47.9–60.9)	65.2 (58.8–71.5)
**HHS Region 9**	2,037	88.2 (84.8–91.1)***	89.5 (86.2–92.3)***	73.2 (68.2–77.9)***	74.1 (69.5–78.2)	73.5 (67.5–79.2)	72.5 (67.8–76.7)	57.8 (53.1–62.7)***	60.7 (55.8–65.7)***
Arizona	437	85.6 (80.3–90.1)***	88.2 (83.1–92.4)***	75.7 (69.3–81.6)***	80.5 (74.3–85.5)	76.2 (65.7–85.4)	69.6 (62.8–75.7)	48.8 (42.2–55.9)***	62.9 (56.3–69.5)***
California	687	88.6 (84.3–92.3)	89.8 (85.6–93.2)***	72.4 (66.1–78.5)***	72.2 (66.3–77.4)	73.7 (66.1–80.7)	73.1 (67.1–78.4)	60.6 (54.5–66.8)***	59.8 (53.6–66.1)***
Hawaii	353	87.6 (83.1–91.4)	88.9 (84.4–92.6)	81.4 (76.1–86.1)	83.9 (78.8–88.0)	73.1 (64.5–81.1)	73.1 (66.6–78.8)	62.7 (56.3–69.2)	69.1 (62.9–75.1)
Nevada	560	87.9 (83.2–91.8)	88.7 (84.0–92.6)	72.1 (66.2–77.8)***	77.8 (72.0–82.6)	70.0 (62.0–77.6)	70.3 (64.6–75.5)	42.5 (37.1–48.3)***	63.1 (57.1–69.0)***
**HHS Region 10**	2,007	90.0 (87.7–92.0)	91.0 (88.8–92.9)***	78.5 (75.4–81.4)***	83.3 (80.7–85.6)	78.9 (74.5–83.1)	75.0 (71.8–77.9)***	63.5 (60.1–66.8)***	69.2 (65.9–72.4)
Alaska	492	83.9 (78.9–88.3)	87.0 (82.1–91.1)	72.6 (66.5–78.3)	73.4 (67.7–78.4)	—^§§§^	71.2 (65.4–76.4)	57.6 (51.7–63.7)	59.8 (53.8–65.9)
Idaho	457	88.9 (84.0–92.8)	88.0 (82.5–92.4)	77.1 (71.0–82.8)	83.9 (78.5–88.1)^†††^	76.8 (66.9–85.5)	75.7 (69.2–81.3)	56.4 (49.8–63.2)	69.4 (63.0–75.6)
Oregon	351	89.0 (84.1–92.9)	90.6 (85.8–94.3)	77.1 (70.7–83.0)	83.1 (77.6–87.5)	80.7 (70.4–89.2)	71.7 (65.0–77.5)	59.9 (53.3–66.6)***	64.3 (57.5–71.0)
Washington	707	91.1 (87.7–93.9)	92.4 (89.3–94.9)	80.1 (75.5–84.3)	84.2 (80.3–87.5)	79.0 (73.2–84.2)	76.8 (72.1–80.9)	67.7 (62.7–72.7)***	72.4 (67.6–77.0)
Range of column values	NA	83.9–95.8	86.5–97.1	69.6–89.4	71.7–86.9	62.7–93.8	62.2–89.4	25.6–80.3	57.8–83.1
**Territory**									
Guam	161	77.3 (67.6–85.8)	—^§§§^	—^§§§^	84.4 (76.1–90.3)	—^§§§^	48.2 (38.4–58.2)	—^§§§^	—^§§§^
Puerto Rico	587	76.2 (70.8–81.3)^†††^	79.8 (74.8–84.5)	65.0 (59.0–70.9)^†††^	72.3 (66.4–77.5)	—^§§§^	61.5 (55.5–67.2)	16.8 (12.8–21.8)	53.5 (47.4–59.8)^†††^
U.S. Virgin Islands^¶¶¶^	58	—^§§§^	—^§§§^	—^§§§^	—^§§§^	—^§§§^	—^§§§^	—^§§§^	—^§§§^

**Abbreviations:** DTaP = diphtheria, tetanus toxoids, and acellular pertussis vaccine; HepA = hepatitis A vaccine; HepB = hepatitis B vaccine; HHS = U.S. Department of Health and Human Services; Hib = *Haemophilus influenzae* type b conjugate vaccine; MMR = measles, mumps, and rubella vaccine; NA = not applicable; PCV = pneumococcal conjugate vaccine; VAR = varicella vaccine.

* The combined seven-vaccine series (4:3:1:3*:3:1:4) includes ≥4 doses of DTaP, ≥3 doses of poliovirus vaccine, ≥1 dose of measles-containing vaccine, the full series of Hib (≥3 or ≥4 doses, depending on product type), ≥3 doses of HepB, ≥1 dose of VAR, and ≥4 doses of PCV.

^†^ Includes vaccinations received by age 24 months (before the day the child turns age 24 months), except for the HepB birth dose (at birth through age 3 days), rotavirus vaccination (by age 8 months), and ≥2 HepA doses (by age 35 months). For all vaccines except the HepB birth dose and rotavirus vaccination, the Kaplan-Meier method was used to estimate vaccination coverage to account for children whose vaccination history was ascertained before age 24 months (also at age 35 months for ≥2 HepA doses).

^§^ Data for the 2020 birth year are from survey years 2021, 2022, and 2023; data for the 2021 birth year are considered preliminary and are from survey years 2022 and 2023 (2024 data are not yet available).

^¶^ Includes children who might have been vaccinated with measles, mumps, rubella, and varicella combination vaccine.

** Includes children who might have been vaccinated with diphtheria and tetanus toxoids vaccine or diphtheria, tetanus toxoids, and pertussis vaccine.

^††^ One dose HepB administered from birth through age 3 days.

^§§^ Includes ≥2 doses of Rotarix (GSK) monovalent rotavirus vaccine, or ≥3 doses of RotaTeq (Merck & Co., Inc.) pentavalent rotavirus vaccine. (If any dose in the series is either RotaTeq or unknown, defaults to the 3-dose series.) The maximum age for the final rotavirus dose is 8 months, 0 days.

^¶¶^ Doses must be ≥24 days apart (4 weeks with a 4-day grace period); doses could have been received during two influenza seasons.

*** Statistically significant decrease in estimated coverage compared with children born during 2018–2019 (p<0.05).

^†††^ Statistically significant increase in estimated coverage compared with children born during 2018–2019 (p<0.05).

^§§§^ Estimate not available because the unweighted sample size for the denominator was <30, or 95% CI half width / estimate >0.588, or 95% CI half-width was ≥10.

^¶¶¶^ Sample size was too small to calculate reliable coverage estimates for the U.S Virgin Islands.

## Discussion

Estimated coverage with most of the routinely recommended childhood vaccines monitored by NIS-Child by age 24 months was lower among children born in 2020 and 2021 compared with coverage among those born in 2018 and 2019. The Healthy People 2030^§§§^ objective to reduce the proportion of children receiving no vaccines by age 24 months (≤1.3%) has been met, but the objectives for ≥1 dose of MMR (≥90.8%) and ≥4 doses of DTaP (≥90.0%) have not. After increasing for several years, coverage with ≥2 doses of influenza vaccine among children born in 2021 declined to 53.4%, more than 10 percentage points below the estimated 63.8% coverage for the 2019 birth cohort. Several studies have documented a negative effect associated with the COVID-19 pandemic on routine pediatric vaccination (*2*–*4*) that could have affected children born in 2020 and 2021. However, analyses of NIS-Child data for children born during 2017–2020 did not identify any consistent or persistent declines in vaccination coverage at the national level and only a few decreases among population subgroups.^¶¶¶^ A recent analysis of trends in parental vaccine hesitancy during 2019–2022 found an increase in hesitancy among parents of children aged 5–11 years after authorization of COVID-19 vaccine, but not among parents of those aged 6 months–4 years (*5*). Parental vaccine hesitancy might be contributing to the low levels of influenza vaccination coverage, due to a higher degree of hesitancy among parents about influenza vaccine compared with other routine childhood vaccines. Hesitancy about influenza vaccine has been observed to be more highly correlated with hesitancy about COVID-19 vaccine than with other childhood vaccines, indicating that parents might perceive influenza vaccine differently than they do other routine non–COVID-19 childhood vaccines (*6*).

This report documents persistent disparities in childhood vaccination coverage by race and ethnicity, poverty status, MSA status, and health insurance status. Vaccination coverage is lower among Black, Hispanic, and AI/AN children, those insured by Medicaid or other nonprivate insurance, children who are uninsured, children living in more rural areas, and children in families with incomes below the federal poverty level. Disparities such as these have been described in other analyses of NIS-Child data (*7*,*8*). In addition to parental vaccine hesitancy, adoption of nonstandard vaccination schedules, and increasing use of religious and philosophical belief exemptions are other significant barriers to the achievement of complete childhood immunization (*9*).

### Limitations

The findings in this report are subject to at least three limitations. First, the low household interview response rates (21%–27% during survey years 2019–2023) and the availability of adequate provider data for only 48%–54% of those with completed interviews during those survey years increases the possibility of selection bias. Such bias might persist to some degree even after survey weighting adjustments, potentially resulting in under- or overestimation of vaccination coverage. Second, potential bias related to noncoverage of households without telephones might also have been incompletely controlled for by the use of weighting in the analysis. If phoneless households are more common among lower income families, the result would be higher observed vaccination coverage. Finally, incompleteness of provider-reported vaccination histories during the most recent survey year could have biased coverage estimates downward. Contractual issues led to a shortened time frame for collecting information from vaccination providers, likely resulting in underascertainment of some administered vaccines. In addition, the vaccination history questionnaire mailed to providers was changed from one to two pages, possibly leading to additional reporting errors owing to lack of familiarity with the new questionnaire format. Assessment of total survey error for the 2023 survey year demonstrated that coverage was underestimated by 2.0 percentage points for ≥1 dose of MMR, 4.3 percentage points for the HepB birth dose, 5.1 percentage points for ≥4 doses of DTaP, and 9.4 percentage points for the combined seven-vaccine series.**** An analysis that evaluated coverage in children from the same monthly birth cohorts who appeared in two different survey years indicated 2–3 percentage point lower estimates based on the 2023 compared with the 2022 samples, indicating a possible change in bias between 2022 and 2023, consistent with the lower provider response rates in 2023.

### Implications for Public Health Practice

Recent decreases in coverage with most of the ACIP-recommended childhood vaccines could lead to a resurgence of vaccine-preventable diseases such as measles, varicella, and rotavirus and their associated morbidity and mortality. During January 1–August 15, 2024, a total of 219 measles cases were reported in the United States, exceeding the number of cases reported annually during 2020–2023 (range = 13 [2020]–121 [2022]). Of the 219 cases in 2024, 190 (86%) were among persons who were unvaccinated or had unknown vaccination status.^††††^ Because children born during or after the period of major disruption of primary care from the COVID-19 pandemic might have missed some vaccinations, providers should review children’s histories and recommend needed vaccinations during every clinical encounter. Addressing financial barriers and other access issues along with vaccine hesitancy and misinformation concerns is important to increasing vaccination coverage and reducing disparities. Higher provider participation in the Vaccines for Children (VFC)^§§§§^ program would help to alleviate some of the financial barriers by increasing access to no-cost vaccines. Other activities that have been found to be effective include reminder/recall systems, implementation of standing orders and clinician prompts, encouraging providers to make strong vaccination recommendations to patients, administering vaccines in alternative settings, and coordination with Immunization Information Systems to identify communities with suboptimal vaccination coverage (*8*,*10*). Implementation of these interventions can increase vaccination coverage, reduce disparities, and bring the nation closer to eliminating vaccine-preventable diseases for all young children.

## Footnotes

*
https://www.hhs.gov/sites/default/files/HHS-Vaccines-Report.pdf

† Additional information about the National Immunization Surveys is available at https://www.cdc.gov/nis/about/index.html. Vaccination against COVID-19 was recommended for children aged 6 months–4 years in June 2022 (https://www.cdc.gov/acip-recs/hcp/vaccine-specific/covid-19.html). Because the recommendation was not in effect until midway through the 2022 data collection year, an accurate estimate of COVID-19 vaccine coverage cannot be calculated from the current NIS-Child data.

§ Vaccination coverage is calculated nationally and for U.S. Department of Health and Human Services regions, states, selected local areas, and the U.S. territories of Guam, Puerto Rico, and the U.S. Virgin Islands. Certain local areas that receive federal Section 317 vaccination funds are sampled separately and included in the NIS-Child sample every year (Chicago, Illinois; New York, New York; Philadelphia County, Pennsylvania; Bexar County, Texas; and Houston, Texas). National estimates in this report exclude U.S. territories.

¶ NIS-Child used a landline-only sampling frame during 1995–2010. During 2011–2017, the survey was conducted using a dual-frame design, with both mobile and landline sampling frames included. In 2018, NIS-Child returned to a single-frame design, with all interviews conducted by mobile telephone.

** The Council of American Survey Research Organizations (CASRO) household response rate is calculated as the product of the resolution rate (percentage of the total telephone numbers called that were classified as nonworking, nonresidential, or residential), screening completion rate (percentage of known households that were successfully screened for the presence of age-eligible children), and the interview completion rate (percentage of households with one or more age-eligible children that completed the household survey). CASRO response rates and the proportions of children with household interviews that had adequate provider data for survey years 2015–2022 are available at https://www.cdc.gov/vaccines/imz-managers/nis/downloads/NIS-PUF22-DUG.pdf.

†† Children with at least one vaccination reported by a provider and those who had received no vaccinations were considered to have adequate provider data. “No vaccinations” indicates that the vaccination status is known because the parent indicated that the child had no vaccinations, and the providers returned no vaccination history forms or returned them indicating that no vaccinations had been administered.

§§ Before 2020, the first dose of HepA was recommended at age 12–23 months, with the second dose administered 6–18 months after the first, depending upon the product type received. During 2020, the recommendation was revised to 2 doses between ages 12 and 23 months, ≥6 months apart, with a catch-up period extending through age 18 years. Because children in this analysis were vaccinated under both recommendations, coverage estimates for both <24 months and <35 months are provided.

¶¶ 45 C.F.R. part 46.102(l)(2); 21 C.F.R. part 56; 42 U.S.C. Sect. 241(d); 5 U.S.C. Sect. 552a; 44 U.S.C. Sect. 3501 et seq.

*** The combined seven-vaccine series (4:3:1:3*:3:1:4) includes ≥4 doses of DTaP; ≥3 doses of poliovirus vaccine; ≥1 dose of measles-containing vaccine; ≥3 or ≥4 doses (depending upon product type) of Hib; ≥3 doses of HepB; ≥1 dose of VAR; and ≥4 doses of PCV.

††† MSA status (a measure of urbanicity) was determined based on household reported city and county of residence and was grouped into three categories: MSA principal city, MSA nonprincipal city, and non-MSA. MSAs and principal cities were as defined by the U.S. Census Bureau (https://www.census.gov/programs-surveys/metro-micro.html). Non-MSA areas include urban populations not located within an MSA as well as completely rural areas.

§§§ No vaccines: https://health.gov/healthypeople/objectives-and-data/browse-objectives/vaccination/reduce-proportion-children-who-get-no-recommended-vaccines-age-2-years-iid-02; MMR: https://health.gov/healthypeople/objectives-and-data/browse-objectives/vaccination/maintain-vaccination-coverage-level-1-dose-mmr-vaccine-children-age-2-years-iid-03; and DTaP: https://health.gov/healthypeople/objectives-and-data/browse-objectives/vaccination/increase-coverage-level-4-doses-dtap-vaccine-children-age-2-years-iid-06.

¶¶¶ https://www.cdc.gov/vaccines/imz-managers/coverage/childvaxview/pubs-presentations/nis-child-pandemic-effects-2018-2021.html.

**** Error profile for the 2023 NIS-Child. https://www.cdc.gov/childvaxview/media/pdfs/2024/09/Error-Profile-2023-NIS-Child.pdf

†††† https://www.cdc.gov/measles/data-research/ (Accessed September 1, 2024).

§§§§ VFC-eligible children include those aged ≤18 years who are Medicaid-eligible, uninsured, AI/AN, or insured by health plans that do not fully cover routine vaccination (if vaccination is received at a federally qualified health center or a rural health clinic). https://www.cdc.gov/vaccines-for-children/about/index.html
